# Phenanthrene Antibiotic Targets Bacterial Membranes and Kills *Staphylococcus aureus* With a Low Propensity for Resistance Development

**DOI:** 10.3389/fmicb.2018.01593

**Published:** 2018-07-17

**Authors:** Bo-Chen Chen, Chang-Xin Lin, Ni-Pi Chen, Cheng-Xian Gao, Ying-Jie Zhao, Chao-Dong Qian

**Affiliations:** ^1^Institute of Molecular Medicine, College of Life Science, Zhejiang Chinese Medical University, Hangzhou, China; ^2^College of Pharmaceutical Science, Zhejiang Chinese Medical University, Hangzhou, China

**Keywords:** dihydro-biphenanthrene, natural product, antibiotic, mode of action, bactericidal effect, membrane-damaging activity, *Staphylococcus aureus*

## Abstract

New classes of antibiotics with different mechanisms of action are urgently required for combating antimicrobial resistance. Blestriacin, a dihydro-biphenanthrene with significant antibacterial activity, was recently isolated from the fibrous roots of *Bletilla striata*. Here, we report the further characterization of the antimicrobial potential and mode of action of blestriacin. The phenanthrene compound inhibited the growth of all tested clinical isolates of *Staphylococcus aureus* including methicillin-resistant *S. aureus* (MRSA). The minimum inhibitory concentrations (MICs) of blestriacin against these pathogens ranged from 2 to 8 μg/mL. Minimum bactericidal concentration (MBC) tests were conducted, and the results demonstrated that blestriacin was bactericidal against *S. aureus*. This effect was confirmed by the time-kill assays. At bactericidal concentrations, blestriacin caused loss of membrane potential in *B. subtilis* and *S. aureus* and disrupted the bacterial membrane integrity of the two strains. The spontaneous mutation frequency of *S. aureus* to blestriacin was determined to be lower than 10^-9^. The selection and whole genome sequencing of the blestriacin –resistant mutants of *S. aureus* indicated that the development of blestriacin resistance in *S. aureus* involves mutations in multi-genes. All these observations can be rationalized by the suggestion that membrane is a biological target of blestriacin.

## Introduction

*Staphylococcus aureus* is an opportunistic pathogen causing a wide range of hospital- and community-acquired infections with high morbidity (Archer, 1998; Al-Mebairik et al., 2016) Diseases caused by the Gram-positive bacterium range from skin infections to life-threatening illnesses, such as septicemia, pneumonia, endocarditis, meningitis, and catheter-related infections due to biofilm formation (Al-Mebairik et al., 2016; Hogan et al., 2016; Ahn et al., 2018). *S. aureus* is also a leading cause of staphylococcal food poisoning because it can produce a wide variety of toxins including staphylococcal enterotoxins and staphylococcal-like proteins (Le et al., 2003; Argudín et al., 2010). The disease caused by *S. aureus* is a worldwide public health issue (Kadariya et al., 2014; Al-Mebairik et al., 2016), and the situation is exacerbated by the emergence of drug-resistant strains, particularly the methicillin-resistant *S. aureus* (MRSA) (Wendlandt et al., 2013; Foster, 2017). Some success has been achieved in the discovery and development of various drugs for the treatment of infections caused by antibiotic-resistant pathogens (Stevens et al., 2004; Ziglam, 2007; Abbas et al., 2017).

Plant-derived antimicrobial agents have received much attention due to their effectiveness against drug-resistant strains, and diverse antimicrobial activities including suppression of virulence factors, antibiofilm activity, anti-persister activity and inhibition of efflux pumps in pathogens (Borges et al., 2015; Mukherjee et al., 2016; Barbieri et al., 2017; Khan et al., 2017). Thousands of phytochemicals have been reported as antibacterial compounds and belong to different classes, such as alkaloids, terpenoids, polyphenols, tannins, and phenanthrenes. Among of them, phenanthrenes are a relatively uncommon class of aromatic metabolites in the plant kingdom, and are mainly found in the Orchidaceae and Juncaceae (Kovács et al., 2008; Tóth et al., 2018). As typical phytoalexins of these plants, phenanthrenes have been shown to possess antimicrobial activities against various pathogens including drug-resistant strains (Kovács et al., 2008; Tang et al., 2008; Yoshikawa et al., 2014; Qian et al., 2015; Tóth et al., 2018). Correspondingly, some phenanthrene-containing plants have been used in traditional medicine for the treatment of infectious diseases (Sun et al., 2010; Namukobe et al., 2011, 2014).

**FIGURE 1:**
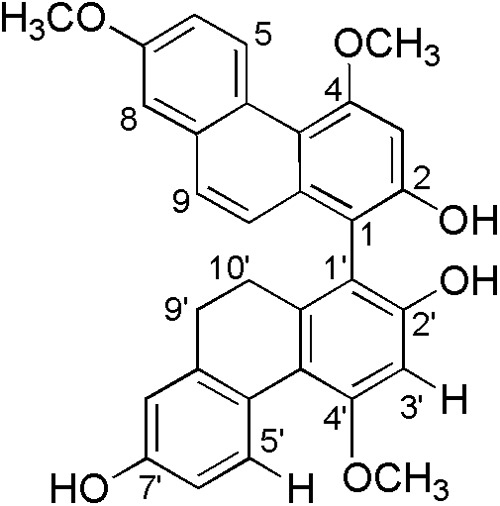
Structure of blestriacin.

*Bletilla striata* (Reichb. f.), a precious medicinal plant, has been used in traditional medicine for the treatment of pneumorrhagia and pneumonophthisis (Sun et al., 2010). Early investigations on the chemical constituents of plant revealed the presence of mono phenanthrenes, dimeric phenanthrenes, and their derivatives (Qian et al., 2015; Guo et al., 2016). The contents of mono phenanthrenes have been found to be higher than those of dimeric phenanthrenes in *B. striata*, although dimers generally have significantly stronger antibacterial activity than the monomers. During a recently screening program of antimicrobial phytochemicals, we found four new 9′,10′-dihydro-biphenanthrenes from the medicinal plant (Qian et al., 2015). One of them, 4,7,4′-trimethoxy-9′,10′-dihydro(1,1′-biphenanthrene)-2,2′,7′-triol, named as blestriacin (**Figure 1**), exhibits significant antibacterial activity against all tested Gram-positive bacteria including MRSA with MICs of 2–4 μg/mL.

Although the antimicrobial activity of phenanthrenes is well documented, their mode of action has not been reported. In this work, we use blestriacin as a tool compound to investigate the antibacterial mechanism of phenanthrenes through a combination of approaches, including minimum inhibitory concentration (MIC)/minimum bactericidal concentration **(**MBC) determination, time-killing assay, growth kinetics analysis, membrane potential estimation, membrane integrity measurement, and genome sequencing analysis.

## Materials and Methods

### Bacterial Strains and Materials

*Staphylococcus aureus* ATCC 29213, *S. aureus* ATCC 43300, and *Enterococcus faecalis* ATCC 29212 were purchased from the American Type Culture Collection. *Bacillus subtilis* 168 was a gift from Mei-Ya Li (Zhejiang Chinese Medical University, Hangzhou, China). Clinical isolates were obtained from patients at the Shaoxing Central Hospital, Shaoxing, China. All bacterial strains used for the activity assay were grown routinely at 37°C on Mueller–Hinton (MH) agar/broth.

Blestriacin was isolated and purified from the fibrous roots of *B. striata* according to the method described previously (Qian et al., 2015). The purity of the compound was determined with HPLC and found to be >95%. The rhizomes of *B. striata* were collected from Tuankou Town, Zhejiang Province, China, and authenticated by Professor ZS Ding. A voucher specimen was deposited in Zhejiang Chinese Medical University with specimen number BS-2012-I. Nisin was purchased from Sigma-Aldrich (St. Louis, MO, United States), and other antibiotics were obtained from Shanghai Yuanye Bio-Technology Co. (Shanghai, China). Propidium iodide (PI) and bis-(1,3-dibutylbarbituric acid) trimethine oxonol [DiBAC_4_(3)] were purchased from BD Biosciences (San Diego, CA, United States) and Enzo Life Sciences (Farmingdale, NY, United States), respectively.

### Determination of MIC and MBC

The MIC of the antimicrobial agents in 96-well microtiter plates was determined by using the broth microdilution method (Guo et al., 2016). Bacteria were seeded at 5 × 10^5^ cells per well in a 96-well plate containing MH broth with varying concentrations of each test sample. Vancomycin, erythromycin, and oxacillin were used as positive controls. Blestriacin was dissolved in dimethyl sulfoxide (DMSO). Sterile MH broth containing 5% DMSO was used as negative control. MBC was determined by subculturing 0.1 mL from each well showing no visible sign of growth in the MH agar. The MIC was defined as the lowest concentration of drug which inhibited visible growth after incubation at 37°C for 20 h, and the MBC was defined as the lowest compound concentration resulting in a ≥3-log reduction in the number of CFU after incubation for 24 h at 37°C (Guo et al., 2016).

### Time-Kill Assay

The time-kill kinetics of blestriacin was determined for four *S. aureus* strains as previously reported (Guo et al., 2016). Briefly, the antimicrobial agent was added to a logarithmic-phase broth culture of approximately 10^6^ CFU/mL to yield concentrations of 1× MIC, 2× MIC, and 4× MIC. The same volume of DMSO was added to the control tube. The culture was incubated at 37°C with shaking for 24 h. One hundred microliter aliquots were removed from each test tube after 0, 0.5, 1, 3, 6, and 24 h of incubation, and serially diluted (1:10) with cold saline. The appropriate dilutions (100 μL) were then spotted on MH agar plates (5 μL per spot), and viable bacteria were quantitated after 24 h of incubation at 37°C. Results were obtained from three independent experiments presented as the average ± standard deviation (SD).

### Growth Kinetics Analysis

Overnight cultures of *Bacillus subtillis 168* were grown in MH broth at 37°C to an OD600 of 0.3. The bacteria were then diluted one-fold in pre-warmed MH broth containing 4× the MIC of the indicated antimicrobial agents. Bacterial growth was monitored by measuring optical density at 600 nm (OD600nm) with a microplate spectrophotometer (Multiscan MK3, Thermo Labsystems).

### Determination of Membrane Potential and Integrity

The effects of blestriacin on the potential of bacterial membrane was tested by flow cytometry with a fluorescent probe DiBAC_4_(3) (Bos et al., 2012). The cells of *B. subtilis* 168 and *S. aureus* 29213 were grown in MH broth to an OD600 of 0.5 and diluted twice with sterilized saline water. After incubation with an antimicrobial agent at 4× MIC for specified time at 37°C, the cells were harvested by centrifugation at 8000 rpm for 5 min and washed twice with sterile saline. The resulting pellets were resuspended in normal saline to approximately 10^6^ CFU/mL. The cells were then stained for 30 min at 37°C with 60 μL of DIBAC_4_(3). Fluorescence was measure with an Accuri C6 flow cytometer (BD Biosciences) with excitation and emission wavelengths of 485 and 520 nm, respectively.

Membrane integrity assays were performed in the same manner as the method described above, except that the fluorescent dye PI was added to a final concentration of 30 μM instead of DiBAC_4_(3). Flow cytometric data were collected with the Accuri C6 flow cytometer with excitation at 490 nm and emission collected at 635 nm. Nisin was used as a positive control for membrane damage and vancomycin as a negative control. The reported results were obtained from three independent cultures.

### Scanning Electron Microscopy (SEM)

For the electron microscopy analysis, overnight cultures of bacteria grown in MH broth was collected by centrifugation (6000 rpm for 5 min at 4°C) and washed three times with sterilized saline water. Bacterial cells present in the suspension containing 10^8^ CFU/mL were incubated with or without blestriacin (32 μg/mL) for 3 h. After incubation, the bacterial suspension was again centrifuged and washed three times with sterilized saline water. A thin smear of bacterial suspension was prepared on a glass slide, air-dried, and fixed with 2.5% glutaraldehyde in phosphate-buffered saline (PBS, 0.1 mol/L, pH7.0) at 4°C overnight. The sample was washed three times with PBS and post-fixed in 1% osmium tetroxide for 20 min, followed by dehydration in gradually increased concentration of ethanol ranging from 50 to 100% (10 min in each stage). The samples were then serially dehydrated in 50, 75, 90, and 100% tert-butanol solutions, followed by vacuum freeze drying for 1 h with a t-BuOH freeze dryer (VFD-21S, Vacuum Device Inc., Japan). The dried cells were coated with gold using a sputter coater (Hitachi E-1010, Japan), and observed under a scanning electron microscope (Hitachi SU8000, Japan).

### Isolation and Analysis of Spontaneous Resistant Mutants

An inoculum of 3 × 10^10^ CFU/mL of *S. aureus* was spread onto MH plates containing different concentrations of blestriacin. All plates were incubated at 37°C overnight and examined after 48 h. Mutational frequency was determined as previously described (Kaul et al., 2013). Individual colonies resistant to blestriacin were isolated and purified from MH agar containing the same blestriacin concentration. The MICs of mutants were confirmed by the broth microdilution method as described above.

### Whole Genome Sequencing and Assembly

Wild type *S. aureus* 43300 (Sa01) together with its drug-resistant mutants Sa02 and Sa03 were subjected to genomic DNA isolation with a ZR Fungal/Bacterial Genomic DNA Extraction Kit (Zymo Research Corp., Orange, CA, United States). Genome sequencing was performed by Sangon Biotech (Shanghai, China). An Illumina MiSeq platform with 2 × 150-bp reads was used for the sequencing. The reads were trimmed, quality-filtered and then assembled by using SPAdes software (Bankevich et al., 2012). BWA/SAMtools (Li et al., 2009; Li and Durbin, 2010) were used for alignment of raw reads and single nucleotide polymorphism (SNP) detection. SNP presented in both mutant strains was confirmed by PCR and Sanger dideoxy-terminator sequencing. Mutations were compared with a reference genome of *S. aureus* MRSA252 (genome NC_002952.2). Sequence similarities were investigated by using the BLAST server at the National Center for Biotechnology Information (NCBI^1^) site. This Whole Genome Shotgun projects have been deposited at DDBJ/ENA/GenBank under the accessions: Sa01 QAJW00000000; Sa02 QAJX00000000; Sa03 QAJY00000000. The version described in this paper is version QAJW 01000000, QAJX01000000, QAJY01000000, respectively.

## Results

### Clinical Isolates of *S. aureus* Are Highly Susceptible to Blestriacin

Blestriacin exhibits significantly anti-Gram-positive bacterial activity against *S. aureus, Staphylococcus epidermidis, E. faecalis*, and *B. subtilis* (Qian et al., 2015). To further evaluate the effect of blestriacin against *S. aureus*, we determined the MICs of the 13 clinical isolates consisting of methicillin-resistant (MRSA) and -sensitive (MSSA) *S. aureus* using a microbroth dilution method. Consistent with previous study (Qian et al., 2015), blestriacin was active against most of the tested clinical isolates with the MICs ranging from 2 to 4 μg/mL, whether the isolate is a methicillin-resistant strain (MICs of Oxacillin > 4 μg/mL) (**Table 1**).

**Table 1:** MICs of blestriacin against clinical isolates of *Staphylococcus aureus.*

Indicator strain	MIC (μg/mL)			
	Blestriacin	Vancomycin	Erythromycin	Oxacillin
*S. aureus* 3211	2	1	>4	>4
*S. aureus* 3304	2	0.5	>4	>4
*S. aureus* 0001	8	1	>4	>4
*S. aureus* 0002	2	1	>4	>4
*S. aureus* 0003	4	1	≤0.5	≤0.25
*S. aureus* 0004	4	2	>4	>4
*S. aureus* 0005	2	1	≤0.5	≤0.25
*S. aureus* 0006	2	1	≤0.5	≤0.25
*S. aureus* 0007	2	1	≤0.5	≤0.25
*S. aureus* 0008	2	1	>4	>4
*S. aureus* 0009	2	1	≤0.5	≤0.25
*S. aureus* 0010	2	1	≤0.5	≤0.25
*S. aureus* 0011	2	1	≤0.5	≤0.25
*S. aureus* ATCC 29213	2	1	0.5	0.125

### Blestriacin Is a Bactericidal Agent

**FIGURE 2:**
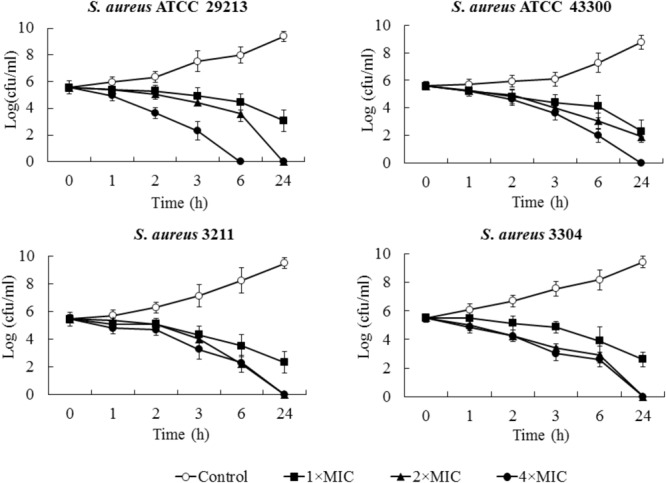
Time-kill curves for *Staphylococcus aureus* treated with different concentrations of blestriacin. The curves are viable cell concentrations plotted against time. Open circles, nondrug control; closed squares, 1× MIC of blestriacin; closed triangles, 2× MIC of blestriacin; closed circles, 4× MIC of blestriacin. MIC, minimum inhibitory concentration. Data represent the mean ± SD (*n* = 3).

Minimum bactericidal concentration test showed that blestriacin exerted bactericidal effect upon *S. aureus* strains with the MBC/MIC ratios of 1–2 (**Table 2**). Interestingly, blestriacin had a MBC/MIC ratio of 1 against *E. faecalis* ATCC 29212, while vancomycin yielded MBC/MIC ratios of >4 against the same strain. To further examine the bactericidal activities of blestriacin against *S. aureus*, we performed killing experiments against two standard strains and two clinical isolates. As shown in **Figure 2**, blestriacin was bactericidal in a dose-dependent manner. No living cell was detected at 24 h for all tested strains treated with 4× the MIC of blestriacin, which is consistent with the bactericidal behavior revealed by the MBC/MIC analysis described above.

**Table 2:** MBCs of blestriacin against *Staphylococcus aureus* and *Enterococcus faecalis.*

Strain and compound	MIC (μg/mL)	MBC (μg/mL)	MBC/MIC
*S. aureus* ATCC 29213 (MSSA)			
Blestriacin	2	4	2
Vancomycin	1	2	2
*S. aureus* ATCC 43300 (MRSA)			
Blestriacin	2	2	1
Vancomycin	2	2	1
*S. aureus* 3211 (MRSA)			
Blestriacin	2	2	1
Vancomycin	1	2	2
*S. aureus* 3304 (MRSA)			
Blestriacin	2	2	1
Vancomycin	2	2	1
*E. faecalis* ATCC 29212			
Blestriacin	2	2	1
Vancomycin	2	>8	>4

### Blestriacin Disrupts Membrane Potential and Integrity

**FIGURE 3:**
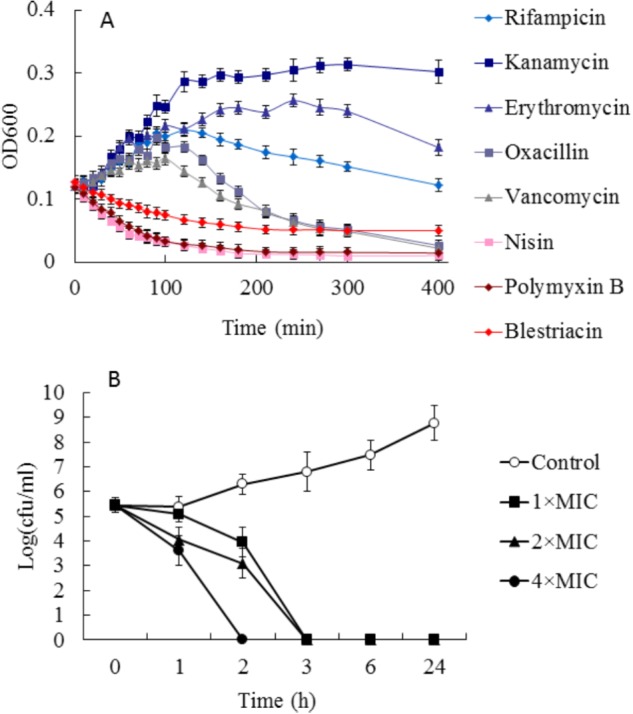
Effect of blestriacin on *Bacillus subtilis* 168. **(A)** Growth kinetics of *B. subtilis* 168 exposed to blestriacin or various antibiotics with known mechanisms of action. Four times the minimal inhibitory concentration (MIC) of the respective compounds were used. **(B)** Killing kinetics of blestriacin against *B. subtilis* 168. Open circles, nondrug control; closed squares, 1× MIC of blestriacin; closed triangles, 2× MIC of blestriacin; closed circles, 4× MIC of blestriacin. The MIC of blestriacin against *B. subtilis* 168 is 2 μg/mL. All experiments were performed in triplicate, and analysis data are presented as the mean ± SD.

To preliminarily investigate the possible mechanism of antimicrobial action of blestriacin, we determined the growth kinetics of *B. subtilis* 168 exposed to blestriacin or various antibiotics with known mechanisms of action (Schneider et al., 2010). The results (**Figure 3A**) demonstrated that blestriacin exhibited kinetic behavior similar to rapidly lytic membrane-active agents (such as polymyxin and nisin) but not to cell wall–interfering agents (such as vancomycin, oxacillin) or antibiotics with transcription (rifampicin), or protein synthesis (kanamycin, erythromycin) as its primary target. Consistently with this, the bacteria killing of blestriacin for *B. subtilis* 168 was rapid at the concentration of 4× MIC, and no living cell was detected within 120 min (**Figure 3B**). Thus, the effect of blestriacin on membrane potential and integrity was investigated.

**FIGURE 4:**
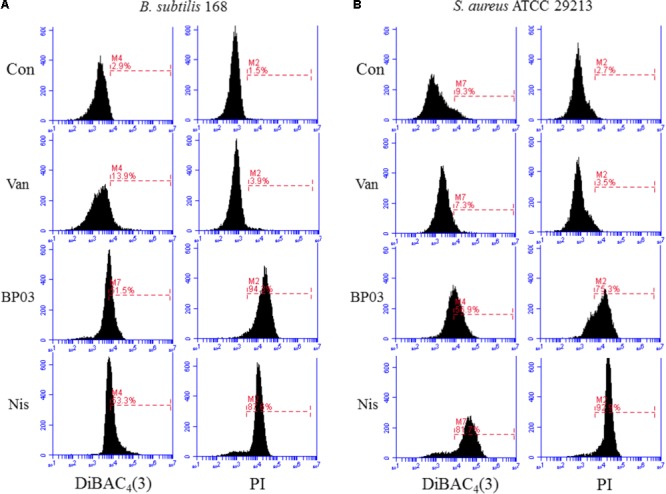
The effect of blestriacin on membrane potential and integrity of *B. subtilis* 168 **(A)** and *S. aureus* ATCC 29213 **(B)**. Representative data from three independent cultures of both strains are shown following exposure to antimicrobial agents at 4× MIC for 2 h, except the incubation time of *S. aureus* ATCC 29213 with blestriacin was 3 h. Vancomycin was used as a negative control and nisin was used as a positive control. Con, no-drug treated control; Van, vancomycin (4 μg/mL); Nis, nisin (20 μg/mL). BP03, blestriacin.

The ability of blestriacin to induce membrane depolarization was evaluated by using the fluorescent dye DiBAC_4_(3), which can enter depolarized membranes and exhibit increased green fluorescence due to its binding to lipid-rich intracellular components (Alam et al., 2015). As expected, the addition of blestriacin or nisin to *B. subtilis* 168 suspensions caused a drastic in fluorescence, indicating that the agent was capable of disrupting the membrane potential of *B. subtilis* (**Figure 4A**). By contrast, the negative-control vancomycin did not alter the membrane potential. The effect of blestriacin on the membrane integrity of *B. subtilis* 168 was monitored using a PI fluorescent probe, which is an indicator of disturbance of plasmatic membrane integrity. Fluorescence measurement showed that blestriacin caused membrane damage of *B. subtilis* 168, which was evident by a great increase in the red fluorescence of cells.

The capability of blestriacin to disrupt membrane potential and integrity of *S. aureus* 29213 was also determined. As shown in **Figure 4B**, when *S. aureus* was treated with blestriacin for 3 h, a drastic increase in fluorescence of DiBAC_4_(3) and PI were both observed compared with the untreated cultures. Interestingly, incubation of *S. aureus* 29213 with 4× MIC of blestriacin for 2 h leads to a weak increase in fluorescence, while incubation of *B. subtilis* 168 with the same concentration of blestriacin for only 0.5 h leads to a great increase in fluorescence of both dyes (data not shown). These results indicated that the membrane of *B. subtilis* 168 is more vulnerable than that of *S. aureus* 29213 to blestriacin.

**FIGURE 5:**
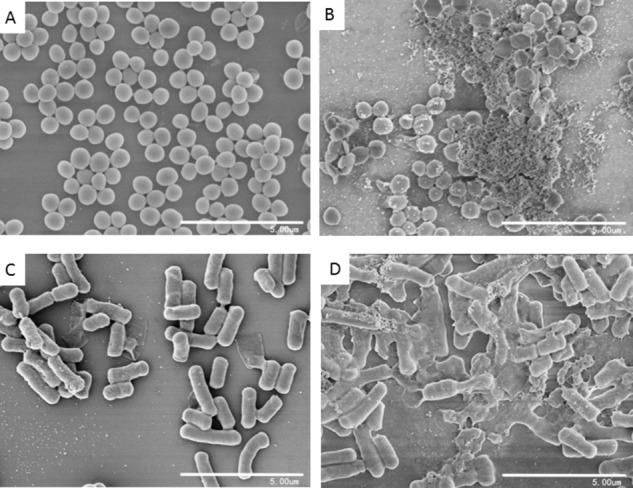
Scanning electron microscopic analysis of bacteria. **(A)** and **(C)**: Represent cells of *S. aureus* ATCC 29213 and *B. subtilis* 168 without treatment at magnifications of 10000×, respectively. **(B)** and **(D)**: Cells of *S. aureus* ATCC 29213 and *B. subtilis* 168 incubated with 32 μg/mL blestriacin for 3 h at magnifications of 10000×, respectively.

To investigate the effects of blestriacin on cell morphology of bacteria, scanning electron microscopic analysis were carried out. As shown in **Figure 5A**, untreated *S. aureus* had smooth and intact morphology. In contrast, numerous lysed cells accompanied by cellular debris were observed in the bacteria treated with blestriacin (**Figure 5B**). Similarly, the antimicrobial agent also induced dramatic morphological changes and structure disruption of *B. subtillus* cells (**Figures 5C,D**).

### Blestriacin Kills *S. aureus* With a Low Spontaneous Resistance Mutation Frequency

To determine the spontaneous resistance mutation frequency of *S. aureus* against blestriacin, we plated *S. aureus* ATCC 29213 at 10^10^ CFU onto MH agar containing 4×, and 8× MICs of blestriacin. No mutant of strain 29213 resistant to blestriacin was observed even when plating on media was conducted with a low dose (4× MIC) of the compound. Then we carried out a study with *S. aureus* ATCC 43300. Blestriacin -resistant *S. aureus* were obtained only by growing the strain on MH agar plates containing blestriacin at 4× MIC. The resistance frequency was determined to be about 2 × 10^-9^. Broth MIC determination of blestriacin -resistant *S. aureus* strains showed twofold increase compared to wild-type bacteria. Interestingly, blestriacin -resistant mutants also exhibited reduced (two-fold) susceptibility to nisin, but showed no changes in sensitivity toward vancomycin and kanamycin.

### Blestriacin Resistance in *S. aureus* Is Associated With Mutations in Multi-Genes

To gain insight into the genetic bases of blestriacin resistance in *S. aureus* ATCC 43300, we isolated and sequenced the genomic DNAs of two independently selected blestriacin resistant strains (Sa02 and Sa03) and parent strain. No significantly change in the growth rates of the two mutants were observed, except the twofold increase in the MIC compared to wild-type *S. aureus*. Whole-genome sequencing revealed six common single-nucleotide polymorphisms in the two mutants, including five nonsynonymous substitutions and one synonymous substitution in coding regions of the genomes (**Table 3**).

**Table 3:** Nucleotide mutations presented in both mutant strains (Sa02 and Sa03) of *S. aureus* ATCC 43300 compared to the wide-type strain.

SNP	Nucleotide change	Predicted amino change	*S. aureus* MRSA252 Locus tag	Predicted gene product
1	T→G	N207K	SAR0206	Maltose ABC transporter substrate-binding protein (Puyet and Espinosa, 1993)
2	A→G	T111A	SAR0210	gfo/Idh/MocA family oxidoreductase (Kanagasundaram and Scopes, 1992)
3	A→T	K225I	SAR2028	Aspartate aminotransferase (Okamoto et al., 1996)
4	T→A	R48S	SAR2257	Putative multidrug efflux transporter
5	C→A	A30S	SAR2480	Transcriptional regulator (Msadek et al., 1990)
6	A→T	V301V	SAR2589	MFS transporter

The Sa02 and Sa03 strains harbored the substitution Arg48Ser in a locus tag, SAR2257, which is annotated as a putative transport protein involved in multidrug resistance (Holden et al., 2004). The locus SAR2257 contains a frameshift after codon 114 in *S. aureus* MRSA252 reference strain but not in the parent and mutant strains of *S. aureu*s ATCC 43300. Four additional nonsynonymous SNPs were identified in open reading frames predicted to encode maltose ABC transporter, oxidoreductase, aspartate aminotransferase and transcriptional regulator. Maltose ABC transporter and oxidoreductase seem to be related to the transport and metabolism of sugars, while aspartate aminotransferase and transcriptional regulator are probably related to the metabolism of amino acids and nitrate regulation, respectively.

## Discussion

In this study, the antibacterial properties of blestriacin, a dihydro-biphenanthrene isolated from the fibrous roots of *B. striata* were investigated. Blestriacin exhibited potent bactericidal activity against *S. aureus*, including MRSA. Several other phenanthrenes from *B. striata* and other plants have also been reported to possess antimicrobial activity against a wide range of pathogens (Takagi et al., 1983; Kovács et al., 2008; Qian et al., 2015). However, most of their MICs exceeded 10 μg/mL. On the basis of the MIC values, which are below 10 μg/mL for the natural compound against *S. aureus* and other Gram-positive bacteria, blestriacin is regarded as an effective antibacterial agent and deserves more attention (Rios and Recio, 2005; Radulovic et al., 2013).

Although phenanthrenes have been studied as antimicrobial agents since the 1980s (Takagi et al., 1983), the mechanism of the action of these compounds has not been investigated. Growth kinetic measurements of *B. subtilis* 168 exposed to various antibiotics showed that blestriacin exhibited kinetic behavior similar to polymyxin and nisin, suggesting blestriacin is a membrane-damaging agent. To test whether blestriacin is a cytoplasmic membrane disruptive agent, we investigated the effect of the compound on membrane potential and integrity. As expected, blestriacin disrupted the bacterial membrane integrity of *B. subtilis* and *S. aureus*, and this was accompanied with the dissipation of membrane potential in two test strains. Consistent with the observation that the bactericidal speed of blestriacin to *B. subtilis* was faster than that of *S. aureus*, the membrane of *B. subtilis* is more vulnerable than that of *S. aureus* against blestriacin. The correlation between the time dependence of antibiotic activity and the effects on membrane integrity further confirmed that blestriacin kills bacterial cells by damaging their cytoplasmic membranes. Consistent with these results, SEM images showed that both bacteria exposed to blestriacin had severely damaged morphology (**Figure 5**).

Pathogens can evolve to acquire resistance to nearly all antibiotics, which is widely associated with treatment failure. The spontaneous mutation rate of bacteria to an antibiotic is closely related to the mechanism of action of a drug used. It is reported that the development of resistance is less frequent for several membrane-active agents than for other antibiotics (Silverman et al., 2001; Hurdle et al., 2011). The spontaneous mutation frequency of *S. aureus* to blestriacin was determined to be 2 × 10^-9^ or less, which is consistent with the finding that blestriacin is a membrane-targeted agent.

Genetic characterization of *in vitro*-derived mutants resistant to an antibacterial agent can provide information about the mechanism of antibiotic resistance as well as the mode of action of this active compound. Whole-genome sequencing of blestriacin -resistant mutants (Sa02 and Sa03) of *S. aureus* and their parent revealed nonsynonymous single nucleotide variants in five open reading frames. One of these genes encoded a putative transporter (SAR2257). Further inspection revealed that the putative protein was similar to the QacA/EmrB multidrug resistance efflux pumps, which were response regulators of two-component systems. The pump QacA conferred resistance to quaternar ammonium cations from *S. aureus* (Rouch et al., 1990), and EmrB protects *Escherchia coil* from uncouplers (Lomovskaya and Lewis, 1992). A unique feature of *emr* consisting of *emrA* and *emrB* is that it seems to confer resistance only to fairly hydrophobic compounds. As blestriacin is a natural phenanthrene with extremely low water solubility, we hypothesized that the putative transporter (SAR2257) is likely to participate in blestriacin efflux from bacterial membranes. However, how the putative transporter participates in blestriacin resistance requires further investigation.

Four additional nonsynonymous SNPs were identified in both mutant strains. Although the role of these mutations is less clear and the predicted functions of these genes seem to be not directly associated with drug resistance, their role in the development of blestriacin resistance in *S. aureus* cannot be ruled out. These gene mutations may help bacteria to better adapt to stress through changes in cell metabolism and transport that optimize the global cellular response to antimicrobial agent exposures. It was recently reported that daptomycin resistance in *E. faecalis* was associated with mutations in eight genes (Tran et al., 2013).

## Conclusion

Overall, our findings demonstrate that blestriacin, a natural phenanthrene, is a unique bactericidal antibiotic. It has a membrane-damaging activity and low propensity to select or induce resistance in *S. aureus*. The development of blestriacin resistance in *S. aureus* involves mutations in multi-genes, which is unlike those agents act upon one target and for which a single point mutation can confer significant resistance. All these observations are consistent with the finding that blestriacin acts as a membrane-damaging antibiotic.

## Author Contributions

C-DQ designed and wrote the manuscript. B-CC, C-XL, N-PC, and Y-JZ performed the experiments and analyzed the data. C-XG provided critical comments on the manuscript.

## Conflict of Interest Statement

The authors declare that the research was conducted in the absence of any commercial or financial relationships that could be construed as a potential conflict of interest.

## Footnotes

1 http://www.ncbi.nlm.nih.gov/BLAST/
